# A Smartphone App for Engaging Patients With Catheter-Associated Urinary Tract Infections: Protocol for an Interrupted Time-Series Analysis

**DOI:** 10.2196/28314

**Published:** 2021-03-23

**Authors:** Robbert Gerard Bentvelsen, Karin Ellen Veldkamp, Niels H Chavannes

**Affiliations:** 1 Department of Medical Microbiology Leiden University Medical Center Leiden Netherlands; 2 Microvida Laboratory for Medical Microbiology Amphia Hospital Breda Netherlands; 3 Department of Public Health and Primary Care Leiden University Medical Center Leiden Netherlands

**Keywords:** catheter-associated urinary tract infections, infection control, patient empowerment, urinary catheter, eHealth

## Abstract

**Background:**

Catheter-associated urinary tract infections (CAUTIs) are the main cause of health care–associated infections, and they increase the disease burden, antibiotic usage, and hospital stay. Inappropriate placement and unnecessarily prolonged usage of a catheter lead to an elevated and preventable risk of infection. The smartphone app Participatient has been developed to involve hospitalized patients in communication and decision-making related to catheter use and to control unnecessary (long-term) catheter use to prevent CAUTIs. Sustained behavioral changes for infection prevention can be promoted by empowering patients through Participatient.

**Objective:**

The primary aim of our multicenter prospective interrupted time-series analysis is to reduce inappropriate catheter usage by 15%. We will evaluate the efficacy of Participatient in this quality improvement study in clinical wards. Our secondary endpoints are to reduce CAUTIs and to increase patient satisfaction, involvement, and trust with health care services.

**Methods:**

We will conduct a multicenter interrupted time-series analysis—a strong study design when randomization is not feasible—consisting of a pre- and postintervention point-prevalence survey distributed among participating wards to investigate the efficacy of Participatient in reducing the inappropriate usage of catheters. After customizing Participatient to the wards’ requirements, it will be implemented with a catheter indication checklist among clinical wards in 4 large hospitals in the Netherlands. We will collect clinical data every 2 weeks for 6 months in the pre- and postintervention periods. Simultaneously, we will assess the impact of Participatient on patient satisfaction with health care services and providers and the patients’ perceived involvement in health care through questionnaires, and the barriers and facilitators of eHealth implementation through interviews with health care workers.

**Results:**

To reduce the inappropriate use of approximately 40% of catheters (currently in use) by 15%, we aim to collect 9-12 data points from 70-100 patients per survey date per hospital. Thereafter, we will conduct an interrupted time-series analysis and present the difference between the unadjusted and adjusted rate ratios with a corresponding 95% CI. Differences will be considered significant when *P*<.05.

**Conclusions:**

Our protocol may help reduce the inappropriate use of catheters and subsequent CAUTIs. By sharing reliable information and daily checklists with hospitalized patients via an app, we aim to provide them a tool to be involved in health care–related decision-making and to increase the quality of care.

**Trial Registration:**

Netherlands Trial Register NL7178; https://www.trialregister.nl/trial/7178

**International Registered Report Identifier (IRRID):**

DERR1-10.2196/28314

## Introduction

### Background

Catheter-associated urinary tract infections (CAUTIs) are the main cause of health care–associated infections and lead to a higher disease burden, increased antibiotic usage, and prolonged hospital stay. Inappropriate placement and unnecessary prolongation of the use of a catheter lead to an elevated and preventable risk of infection.

The smartphone app Participatient has been developed to involve patients in communication and decision-making related to catheter use with the aim to overcome unnecessary (long-term) catheter use and prevent CAUTIs. Participatient can potentially empower patients to bring about sustained behavioral changes and prevent infections.

### Previous Studies

Participatient was developed at Dutch Hacking Health Leiden 2016 as a prototype smartphone app to prevent CAUTIs by involving patients in health care–related decision-making. The jury awarded the Participatient development team with the first prize nationwide for developing a patient involvement interface with an innovative design for infection prevention.

Participatient engages patients by providing them with personalized information regarding the appropriateness of their catheter, along with other medical and admission-related information.

The prototype was further developed in collaboration with patients, hospital staff, social scientists, engineers, eHealth experts, infection control professionals, and clinical microbiologists. We invited patients and staff in clinical wards to express their needs and concerns and to test, rate, and provide feedback on the initial versions of Participatient and its content.

Through 3 rounds of improvements, based on 5-10 interviews with patients [1], and 2-4 nurses per round, we developed the final version of the app. The interviews were based on the technology acceptance model [2] based on the usefulness and the ease of use of the app and its features. Furthermore, we majorly focused on patients’ skills in using technology and their eHealth literacy in order to increase the usability further [3,4]. The app was adjusted to the patients’ requirements by including additional information, although this was optional so as to not cause inconvenience to those with eHealth experience.

This led us to (1) generate iconic graphics to clarify text, (2) use visual feedback intermezzos containing motivational text or an explanation of the results, (3) use plain language adjusted to the level of understanding of patients in general, (4) develop clickable and thus optional instructions in the questionnaire, and (5) disseminate practical information, which was most valued by patients in the hospital wards. The patient information leaflet was digitized in the app.

After the development phase, a proof-of-concept study was conducted at a clinical ward at Leiden University Medical Center (LUMC), in which the technical and interactive aspects of the app were tested. All patients admitted to this ward were invited to download the app for use during their hospital stay. The app was introduced to the patients by the nurse who managed patient admission. We actively supported app use and assessed and adjusted the app through feedback options and a questionnaire. Users expressed positive opinions about the app’s purpose and design. We received some valuable feedback regarding the app’s content, which was generated using the Catheter Check content module. Users scored the app with 5 out of 5 stars.

### Participatient Content

**Figure 1:**
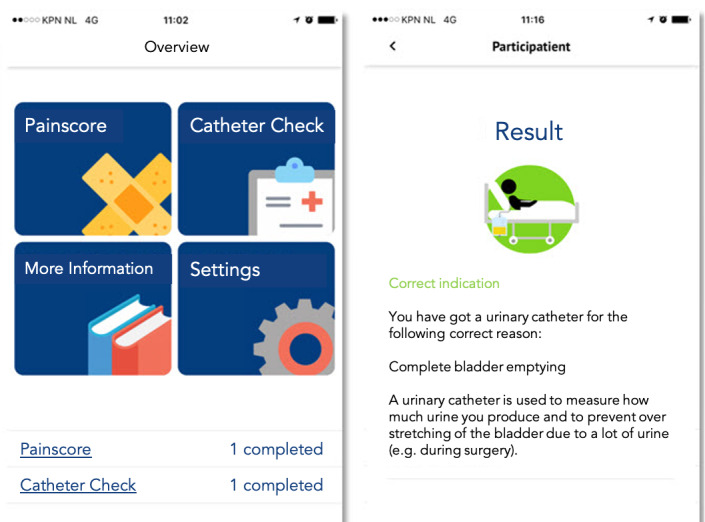
The Participatient app content.

During initial app use or “on-boarding,” the patient is asked to provide information regarding their ward of admission, gender, age group, and previous internet or app usage. This information is used to provide directed information regarding their admission. The app can be downloaded from the Apple App store and Google Play store on a patient’s smartphone or tablet device and comprises four content modules: Catheter Check, Admission Information, Pain Score, and Feedback. Finally, a Settings menu is included in the app (Figure 1).

Catheter Check is the catheter module, in which the appropriateness of catheter use is assessed by answering 8 questions. This yields a score in accordance with nationwide and worldwide criteria [5,6]. The result is displayed with personalized suggestions, promoting shared decision-making by motivating the patient to initiate dialogue with medical or nursing staff on the appropriateness of the presence of the catheter. Through daily reminders, patients are motivated to regularly check the indication of their catheter.

The Admission Information module comprises general medical information regarding infections, catheters, and the prevention of health care–associated infections for patients, but also practical information regarding the ward. From the patient feedback in the development phase, we learned that practical details including the visiting hours and telephone numbers of the ward were the most highly desired information. Participatient includes an information module with ward-specific information. In the proof-of-concept study, we used the patient information available in the ward’s paper-based records and digitized it in the app to include images and links.

The Pain Score module yielded an adopted pain score that accounts for various factors including mobility, medication, and myths and facts on pain relief. This module motivated patients to ask for adequate pain medication and medication to combat side effects including nausea when needed. Pain score evaluation is not a part of this study proposal. Nonetheless, this module has been developed for better pain registration, advice and education on side effects, and motivation of patients to seek better pain management, leading to an enhanced health care experience. Through daily reminders, patients are motivated to regularly score their need for pain medication.

The Feedback module contains an 8-question survey on patient satisfaction with the app and a link for email communication with the researchers.

In the Settings module, the daily reminders can be adjusted or turned off, and basic demographic characteristics, including gender, age group, and specialty and ward of admission, can be visualized and modified.

Participatient is available free of cost to all patients in the participating hospitals. The costs of adjustment and deployment are covered by the research team. App download and usage are limited by a code that is provided to the patients on admission.

Through the Admission Information and Pain Score modules in the app, users can avail of information and advice on other useful topics in addition to the catheter. In the development and proof-of-concept phases, users appreciated the additional functions of the app and indicated that this further motivated them to continue using the app throughout their hospital stay.

### Study Objective

During the implementation study, we aim to investigate whether the involvement of patients in infection prevention through an eHealth tool is effective and sustainable. Our primary objective is to reduce catheters without an inappropriate indication in clinical wards by 15% by implementing Participatient.

Our secondary objectives are as follows: (1) to reduce CAUTIs; (2) to increase patient satisfaction with health care, their involvement in health care, and their trust in physicians; (3) to measure patient satisfaction with the usefulness and ease of use of Participatient and to optimize the app on the basis of these outcomes; and (4) to obtain analytical information regarding the use of various modules of the app and feedback from users for its further development and to make it available for extended use in preventing health care–associated infections or CAUTIs in primary and long-term care.

## Methods

### Overview

The app will be introduced in various clinical wards at 4 hospitals participating in the study. The app will be adjusted in accordance with the requirements of each medical center. Through a stepwise approach, we will launch the study at multiple locations.

The study will be implemented at clinical wards with a training session for the nurses in each ward, which will include a “kick-off” day that involves a demonstration of the app and an interactive session with the research team. We will provide information and instructions for downloading and installing the app on leaflets, which are normally provided to patients upon admission. Posters and flyers with infographics elucidating the risk of nosocomial infections and the study are provided to the participating clinical wards.

In each ward, we shall assess the willingness among and potential barriers to patients and health care workers. Before launching the app at each ward, it will be adjusted and extended to contain local information, protocols, and links to relevant websites of the 4 participating hospitals.

At each ward, an ambassador, with an affinity for the study, is recruited from among the nursing staff to provide peer support. This has been tested and found to be very useful in the proof-of-concept phase of this study. For active engagement of the ward staff, we included a regular support day in the ward for technical and medical troubleshooting during the implementation phase and the postintervention phase.

This is an interrupted time-series (ITS) analysis with the implementation phase between the pre- and postintervention survey. After the implementation phase, we will record feedback from the wards with regard to the prevalence and indication of catheters during the previous surveillance period. This “mirroring” technique is used in intervention studies and patient care to motivate subjects to facilitate further improvements. This would foster awareness and help reduce the use of catheters. Integration of an app in the health care routine is a complex intervention, with the implementation process itself also adding to the intervention. We will use the Trials of Intervention Principles Framework [7] to evaluate the overall effect of app implementation. During the postintervention surveys, we will report data on app use per department. After the postintervention surveys, we will report the surveillance data per department and study site.

The Participatient website [8] contains general information regarding the study. For the implementation phase, the website will be revised and updated. After consulting patients and wards, we aim to provide relevant information from hospital admission to nosocomial infections. The relevant information provided in the app will be made available on the website.

### Participating Hospitals

The following hospitals participated in this study: clinical wards of the LUMC (Leiden, the Netherlands), with the introduction of the app as main intervention; clinical wards of the Haaglanden Medical Center (The Hague, The Netherlands)—a regional general hospital—with the introduction of the app as the main intervention; clinical wards of the department of internal medicine and neighboring specialties of the Amsterdam University Medical Center (Amsterdam, The Netherlands) and the Spaarne Gasthuis (Haarlem, The Netherlands) as the second hospital from the Reduce the Inappropriate Use of Urinary and Intravenous Catheters study, with the app as an addition to the intervention bundle.

### Patient Recruitment

All patients in the participating wards, aged ≥18 years, or their family members will be able to download the app from the Apple App store and Google Play store and use it on their personal smartphone or tablet device (Android, iPhone, or iPad devices). The patients and staff are included in each step of the process in accordance with the Patients Included charters on patient information resources.

#### Ethics Statement

All data are processed anonymously. The studies are and will be conducted in accordance with the tenets of the Declaration of Helsinki, and all procedures involving patients have been approved by the Medical Ethical and Institutional Review Board of the LUMC. Before conducting the interviews, distributing the questionnaires, or using the app, informed consent will be obtained, either in the verbal or written format in the case of interviews and paper-based questionnaires or in the digital format in the app before initial use, from all users to anonymize the data for analysis.

For clinical data on the improvement of care quality, consent will be obtained as described previously [6]. Patients are offered the option to opt out of the study at any time by being discharged by the treating physician, as stated in the hospital admission information [9].

#### Study Design

**Figure 2:**
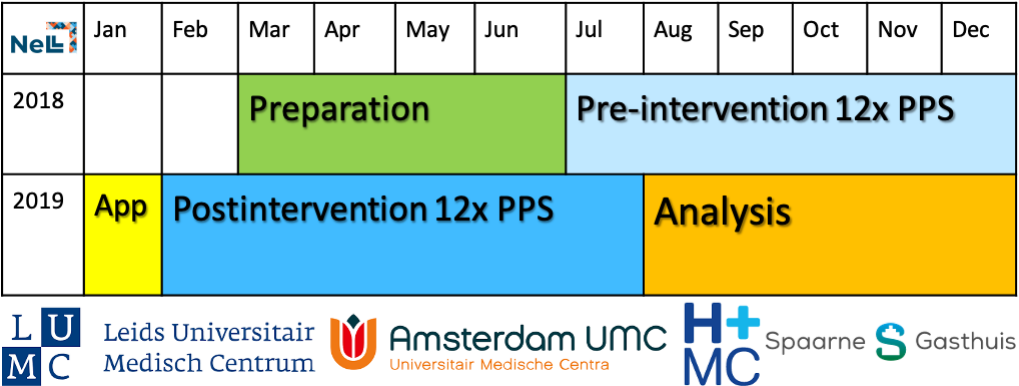
Interrupted time-series analysis design with point-prevalence surveys (PPS).

We will conduct a multicenter ITS consisting of a pre- and postintervention point-prevalence survey distributed among the participating wards to investigate the efficacy of the app in reducing the inappropriate use of catheters (Figure 2) [10].

We will conduct the ITS as described previously [11]. Clinical data will be collected every 2 weeks for 6 months in the pre- and postintervention periods.

All admitted patients in the participating wards will be included in the point-prevalence survey regardless of app use, catheter presence, or urinary tract infection (UTI). A local study code will be generated for each patient for those with a catheter or a UTI. This code is a pseudonym to verify the collected data and rectify as required. This code will remain in the local hospital and only be used during the study.

During the point-prevalence surveys, the following data will be collected: demographic factors including gender, department and specialty of admission, age in decennia, and the date of admission; catheter use including the date of insertion of the catheter, indication of the catheter on insertion, and the indication of the catheter at the time of the survey; and UTI episodes occurring at the time of the survey and if a catheter was associated with them.

The presence and indications for catheter use will be extracted from the (electronic) medical records, nurses’ lists, and observations of the admitted patients. On the day of measurement, the indication of the catheter and the necessary patient variables will be collected.

#### Measurement of Patient Satisfaction

To investigate the secondary outcomes, namely the impact of the app on patient satisfaction with health care services and providers and the patients’ perceived involvement in health care, the following validated instruments will be included in the questionnaires. Paper-based questionnaires on satisfaction with care, trust in the physician, and communication will be distributed. These data will be collected along with additional data including gender, department and specialty, and age group. The questionnaire on patient satisfaction with the app will be administered through the app itself.

Satisfaction with care will be measured using the items of the Quality of Care Through the Patient’s Eyes (QUOTE) questionnaire [12]. In total, 6 items will be used to measure patient satisfaction with the physician and nurse.

Trust in the physician will be measured using Trust in physicians_short form (TRIP_sf), which is based on the Cologne Patient Questionnaire scale to evaluate the patients’ trust in physicians, which measures different aspects of trust during physician-patient interactions [13].

Self-efficacy during patient–health care provider communication will be measured with the 5-item version of the Perceived Efficacy in Patient-Physician Interactions (PEPPI-5) questionnaire, which assesses the subjective sense of patients’ confidence when interacting with their physicians and patient involvement in health care [14,15].

To assess patient satisfaction with the app, a short 8-item evaluation questionnaire on the ease of use, time investment, usefulness, and perceived effect of the app will be used. Patient feedback will be requested through leaflets, and there will be a dedicated button on the home screen in the app.

Moreover, sociodemographic factors and general previous usage of eHealth or apps among patients will be measured [3,4] to determine the presence of patient features that predict the use and satisfaction with the app. To determine whether the app is fit for use by a wide range of users, user information will be requested upon on-boarding (initial use).

We will evaluate the health care workers’ experience with the implementation of shared eHealth-related decision-making on the wards before and after comparison interviews to detect barriers and facilitators [16] in the wards at each center.

#### Analytical Data on App Use

Data on app usage and data obtained from the end users will be collected via the app and accompanying questionnaires. Usage data (analytics) on the number of downloads, the page views, and continued app usage will be collected. These data are traced to the users per ward, gender, internet or app experience, and age group, although they are not traceable to an individual level (privacy by design). User-provided data comprise the responses to the catheter check, pain score, and the use of the Admission Information module.

#### Data Management Plan

**Figure 3:**
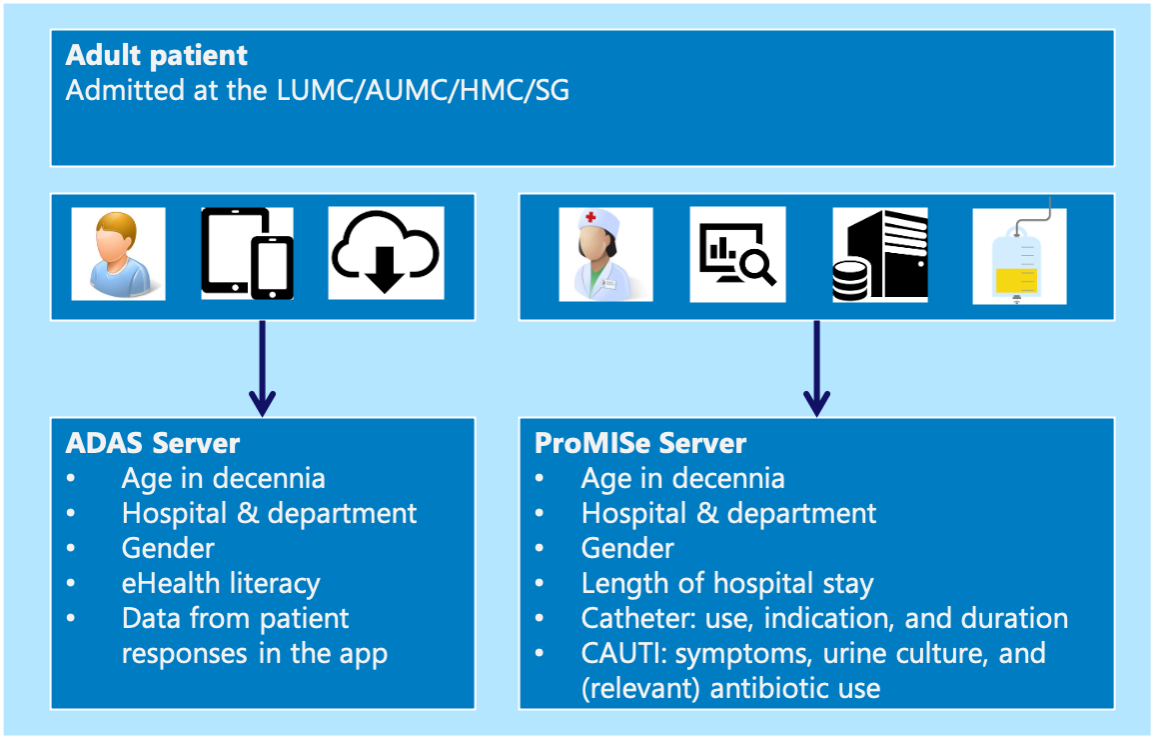
Data collection and secure storage at the LUMC on ADAS and ProMISe servers. CAUTI: catheter-associated urinary tract infection, AUMC: Amsterdam University Medical Center; HMC: Haaglanden Medical Center; LUMC: Leiden University Medical Center; SG: Spaarne Gasthuis.

All data entered via the App will be stored with an anonymized ID in the app and on secure data management servers ADAS and ProMISe, which are located at the LUMC and managed by the Advanced Data Management section and are ISO 27001 certified. Data files used for analysis will be stored in the safe network storage facility DataSafe (Figure 3).

Data will be collected by the research physician (RB) at the LUMC. Participants will respond to the questionnaires. After completion of a form, the app will contact the ADAS server of the LUMC and transmit the entered data, which is then stored as message files. These files are validated and transformed into requests for automatic data entry into the ProMISe server. The ProMISe server provides the functionality for data management, including data export to SPSS for statistical analysis, wherein an SPSS export will be performed, and these files will be stored in protected network storage (DataSafe) with access limited to the investigators.

### Analysis

#### Sample Size

The sample size is based on our objective of reducing inappropriate catheter use by 15%, with a power of 80% and a Cronbach *α* of .05. We extracted data on the incidence of inappropriate catheter use from previous studies in similar health care systems, which was approximately 40% for catheters [17,18]. We aim to collect 9-12 data points from among 100 patients per survey date per hospital [19,20]. On designating a particular day to obtain measurements every 14 days at each center, a 5-month period before and after the measurements is required. We intend to carry out surveys for 6 months and to collect 12 data points. At the LUMC, the prevalence of catheter use is approximately 30% among hospitalized patients, based on our pilot survey. Thus, 1200 patients are required per period, of whom catheters were used for 400 patients, and catheter usage being inappropriate among 160 patients. On correlating for 10%-15% of the missing data, the sample size is set to 1320-1380 patients in the pre- and postintervention groups. We intend to include this number of patients at each hospital to analyze the effect of the interventions at each medical center.

#### Statistical Analysis

We will conduct an ITS analysis, similar to the one performed previously [21,22]. ITS is considered a strong study design when randomization is not possible and can thus be used to investigate causal effects with an observational “natural experiment” approach. The Cochrane collaboration guidelines for ITS analyses will be used with an autoregressive integrated moving average model [23]. Because such a model relies on linearity, we will assess the stationarity of the mean and variance with time through differencing [24,25]. The primary analysis involves a comparison of inappropriate catheter usage before and after Participatient implementation. Because other changes in catheter usage could affect our outcomes over time, we will adjust the data for potential confounders, autocorrelation, and the underlying secular trend. Subgroup analyses will be performed on the basis of risk factors for catheters and UTIs, including the ward of admission, age group, internet or app use, and gender. Analyses will be conducted using SPSS (version 24, IBM Corp).

We will use figures to visualize trends and the impact of the intervention. We will present the difference in unadjusted and adjusted rate ratios with a 95% CI. Differences will be considered significant when *P*<.05. All analyses, including subgroup analyses, will be predefined in an analysis plan before their performance.

For questionnaire assessment, we will use descriptive statistics and compare patient satisfaction with health care, their involvement in care, and their trust in physicians, before and after implementing the app. Data on patient satisfaction regarding the usefulness and ease of use of Participatient will be analyzed using descriptive statistics and will be used for subsequent rounds of app improvement after the study.

## Results

Based on our objective of reducing the inappropriate use of approximately 40% of catheters by 15%, we aim to collect 9-12 data points from among 70-100 patients per survey date per hospital. We will conduct an ITS analysis, which is considered a strong study design when randomization is not feasible. We will present the difference in unadjusted and adjusted rate ratios with a 95% CI. Differences will be considered significant when *P*<.05.

## Discussion

This protocol describes the objectives, design, intervention, and survey methods for the “Patient Engagement Counter Catheter-associated urinary tract infections with an App” study and aims to prevent the inappropriate and prolonged use of catheters at acute care facilities.

A potential limitation of an ITS analysis is the lack of a control group. However, this quasi-experimental design is considered to be among the most effective and powerful designs when randomization is not feasible. Another limitation of this protocol is its inability to evaluate the impact of app use on an individual level. However, the intervention stimulates communication and creates awareness regarding the risks of inappropriate catheter use among all ward staff through patient engagement, thus benefiting all patients in the ward.

Thus far, patient involvement in infection prevention has been undervalued and unused as a means to improve the quality of care. By sharing reliable information and daily checklists to patients via an app, we can provide them a tool to involve them in the management of catheter use. Thus, inappropriate catheter use is expected to be better noticed and discouraged, and the risk of CAUTIs could be reduced.

## Appendix

Multimedia Appendix 1 Reviewer Comments B.2017.01BA0 from The Netherlands Organisation for Health Research and Development (ZonMw): project 522004007, dossier number 50-52200-98-559.

Multimedia Appendix 2 Reviewer Comments B.2017.01BA1 from The Netherlands Organisation for Health Research and Development (ZonMw): project 522004007, dossier number 50-52200-98-559.

Multimedia Appendix 3 Reviewer Comments B.2017.01BD2 from The Netherlands Organisation for Health Research and Development (ZonMw): project 522004007, dossier number 50-52200-98-559.

## Abbreviations

CAUTI: catheter-associated urinary tract infection
ITS: interrupted time series
LUMC: Leiden University Medical Center
UTI: urinary tract infection
