# Supplementary material for: A Smartphone App for Engaging Patients With Catheter-Associated Urinary Tract Infections: Protocol for an Interrupted Time-Series Analysis
Source: JMIR Res Protoc. 2021 Mar 23;10(3):e28314. doi: 10.2196/28314 (PMC8086777; doi:10.2196/28314)
Supplement: Multimedia Appendix 1 [file resprot_v10i3e28314_app1.pdf]

Subsidieprogramma / Subsidy programme	:	**Infectieziektebestrijding 2014-2017**
Dossiernummer / Dossier number	:	**50-52200-98-559**
Aanvrager / applicant	:	**Prof. dr. N.H. Chavannes MD PhD**
Projecttitel / Project title	:	**Reducing the risk of catheter-associated urinary tract infections via a smartphone application for patients – Participatient**
Beoordelingscode / Assessment code	:	**B.2017.01BA0**

1. General

Please provide considerations which contributed to your assessment of each of the assessment criteria.

Note that we will send your assessment to the applicant in anonymised form. He or she will then have an opportunity to respond. We would therefore urge you to avoid making any references to yourself in your reviewer's report.

2. Criteria

Legenda: E (Excellent,), VG (Very good), G (Good), S (Sufficient), U (Unsatisfactory)

2.1 Objective, problem definition and assignment

E	VG	G	S	U
	X			

Objective, problem definition and assignment Consider the following factors:

- how clear and specific the objective is;
- how clear and verifiable the problem definition/assignment is and whether it is consistent with the objective;
- the value added to existing knowledge or practice;
- the theoretical or empirical evidence presented in support of the problem definition/assignment.

The objective of the research is clearly presented – to test a mobile app designed to increase patient involvement in urinary catheter use to reduce catheter-related urinary tract infections with patients in two networks of hospitals using an quasi-experimental interrupted time series design.

The applicant provides evidence that the research will address a large problem, i.e., health care-associated infections, that if reduced will have substantial benefits to patients and the health care system. Also, evidence is provided that indicates a large proportion of urinary catheters are used unnecessarily, adding to the risk of infection.

Evidence is provided that interventions with health care providers have had inconsistent success and that patients are not actively involved in decisions regarding urinary catheters. The mobile app is designed to address this latter gap by helping patients become involved in urinary catheter decisions. If successful, the research will add substantial value to existing practices in hospitals to prevent urinary tract infections.

The basis for this research is primarily the empirical evidence that the problem is large, past interventions have varied in success, and patients have been activated to play a role in decisions regarding infection prevention. The mobile app also follows guidelines for urinary catheter use in its Catheter Check.

Regarding patient involvement, the applicants suggest that past interventions that have involved patients in hand hygiene provide evidence for the expectation that they are willing to be involved in decisions regarding urinary catheters. This conclusion is weak because hand washing is not an invasive procedure and may not involve high-status health care workers such as physicians which patients may be reluctant to confront and correct even if the mobile app tells a patient a urinary catheter is not indicated.

2.2 Strategy

E	VG	G	S	U
X				

Consider the following factors:

- clarity;
- adequacy in terms of problem definition/assignment;
- adequacy of chosen methods and analyses;
- adequate random sampling and power calculation;

- the way in which the strategy reflects the factors gender, age, ethnicity and/or other characteristics relevant to the objective;
- degree of collaboration with intermediate and/or ultimate target group (the patient/client perspective).

With an implementation project:
- analysis of the context in which implementation is to take place;
- extent to which target groups are mentioned;
- a good mix of implementation activities;
- analysis of factors facilitating or hampering those activities;
- participation of stakeholders;
- prospect of structural incorporation in system;
- adequacy of process and effect evaluation design.

The primary and secondary aims of the research are clear and achievable.

The mobile app to empower patients in decisions regarding urinary catheters has already been developed and is available for this project. User-centered design methods were used to iteratively develop the mobile app and obtain extensive input from various stakeholders, e.g., patients, hospital staff, social scientist, engineers, e-Health experts, infection control professional and clinical microbiologists. The mobile app content is clearly and fully described.

It is not clear whether the mobile app has completed proof of concept testing or this will be done in the proposed study. If it is already completed, results of the proof of concept testing would strengthen the evidence for implementing and testing the mobile app. The applicants also mention app improvement rounds but it is not clear if and when those will occur.

The interrupted time-series design is a quasi-experimental design which will provide strong evidence on the effectiveness of the mobile app. This is an appropriate design for testing the mobile app in actual practice in hospitals. Multiple measures are planned both pre- and post-intervention and intention-to-treat analyses appear to be planned. The analysis plan uses the appropriate ARIMA methods for evaluating an interrupted time-series design.

The applicant describes how the mobile app will be introduced to hospital staff and patients in the interrupted time series design. Plans to recruit an “ambassador” on each ward to support mobile app use will help to ensure its use.

The patient population is described in terms of its location in the participating hospitals but limited information is provided on the inclusion and exclusion criteria beyond age and ability to download and use a mobile app. It is not clear if all patients will be on wards where they are likely to be given a urinary catheter.

The sample size is clearly stated and power calculations are provided confirming the study will have 80% power.

The outcome measures are strong and will be obtained from electronic medical records and observation of patients. Patients will also report on their satisfaction with care and care providers and involvement in care. Data on the use of the mobile app also will be collected. There is a data management plan. All are appropriate to achieve the objective of the research.

The goal of this project is to implement it in two networks of hospitals with hospital staff and patients. If successful, the applicant plans to implement it in other hospitals or other regions.

The main implementation activities are comprised of traditional academic communication, through peer-reviewed publications, presentations to health professionals, and distribution as an open source app. Less attention is paid to considering how to convince hospital staff and patients to use the mobile app to achieve this implementation beyond the research.

The applicants have considered a number of potential strengths and weaknesses to the strategy and addressed them appropriately.

2.3 Project group

E	VG	G	S	U
X				

Consider the following factors:
- relevant expertise;
- familiarity with area in question;
- prior activities and products

The research team has the expertise, skills and experience to conduct the study in the areas of microbiology, infection control, e- and m-Health, patient involvement, social sciences, and biostatistics.

Dr. Chavannes is a senior researcher with considerable experience leading project teams.

The creator of the mobile app, Dr. Bentvelsen, is on the team.

2.4 Feasibility

E	VG	G	S	U
X				

Consider the following factors:
- will it be possible to achieve the objective(s) using this strategy?
- availability of facilities/staff;
- realistic phasing and timetable.
- factors which may positively or negatively impact the feasibility;
- collaboration with relevant stakeholders and intermediate target groups.

Feasibility is high and all objectives of the research should be achievable.

The mobile app already exists.

The team has access to the hospital networks to recruit the patients.

The timeline for the project is ambitious but achievable given the support of the hospital networks and the existing mobile app.

The group has collaborated with many of the stakeholders during development of the mobile app and they should be available to help achieve the research.

2.5 Overall quality assessment

E	VG	G	S	U
X				

The potential impact of this research is large and the proposed research activities are very achievable. The mobile app already exists, follows existing guidelines, and can be quickly implemented. Implementation procedures for the study are well-described. The primary and secondary outcome measures are appropriate. The quasi-experimental interrupted time-series design is very appropriate for the research objective. The study methods are very strong. The analysis plan is state-of-the-art. If successful, the mobile app will fill a large gap in infection control and should be capable of being disseminated widely to other hospitals. Remaining weaknesses are minor and easily addressed by the applicants. The study has a short timeline that will mean the evidence for the effectiveness of the mobile app will be available quickly.

3. Budget

Legenda: TH (Too high), R (realistic), TL (too low)

3.1 Budget

TH	R	TL
	X	

The budget is appropriate for the scale of the project and the activities planned.
