# Supplementary material for: A Smartphone App for Engaging Patients With Catheter-Associated Urinary Tract Infections: Protocol for an Interrupted Time-Series Analysis
Source: JMIR Res Protoc. 2021 Mar 23;10(3):e28314. doi: 10.2196/28314 (PMC8086777; doi:10.2196/28314)
Supplement: Multimedia Appendix 2 [file resprot_v10i3e28314_app2.pdf]

Subsidieprogramma / Subsidy programme	:	**Infectieziektebestrijding 2014-2017**
Dossiernummer / Dossier number	:	**50-52200-98-559**
Aanvrager / applicant	:	**Prof. dr. N.H. Chavannes MD PhD**
Projecttitel / Project title	:	**Reducing the risk of catheter-associated urinary tract infections via a smartphone application for patients – Participatient**
Beoordelingscode / Assessment code	:	**B.2017.01BA1**

1. General

Please provide considerations which contributed to your assessment of each of the assessment criteria.

Note that we will send your assessment to the applicant in anonymised form. He or she will then have an opportunity to respond. We would therefore urge you to avoid making any references to yourself in your reviewer's report.

2. Criteria

Legenda: E (Excellent,), VG (Very good), G (Good), S (Sufficient), U (Unsatisfactory)

2.1 Objective, problem definition and assignment

E	VG	G	S	U
X				

Objective, problem definition and assignment Consider the following factors:
- how clear and specific the objective is;
- how clear and verifiable the problem definition/assignment is and whether it is consistent with the objective;
- the value added to existing knowledge or practice;
- the theoretical or empirical evidence presented in support of the problem definition/assignment.

This project focuses on a clear problem - inappropriate use of indwelling urinary catheters, with a clear and clinically important and feasible target (15% reduction) as the primary aim, as well as interesting secondary aims (CAUTI reduction, increase in patient satisfaction in care, as well as satisfaction with the app, as well as data collection to inform implementation in other settings.
My only question is why there is a focus on "(long-term)" use of catheters as the principal goal, when the described project seems more focused on reducing short-term use of temporary catheters in the acute-care setting where average hospitalization is <=5 days, rather than long-term use. I suggest clarifying your definition/understanding of "long-term use" in this section and throughout.

2.2 Strategy

E	VG	G	S	U
		X		

Consider the following factors:
- clarity;
- adequacy in terms of problem definition/assignment;
- adequacy of chosen methods and analyses;
- adequate random sampling and power calculation;
- the way in which the strategy reflects the factors gender, age, ethnicity and/or other characteristics relevant to the objective;
- degree of collaboration with intermediate and/or ultimate target group (the patient/client perspective).

With an implementation project:
- analysis of the context in which implementation is to take place;
- extent to which target groups are mentioned;
- a good mix of implementation activities;
- analysis of factors facilitating or hampering those activities;
- participation of stakeholders;
- prospect of structural incorporation in system;
- adequacy of process and effect evaluation design.

The project is initially described as implementing an app that patients use to better understand urinary catheter risks/benefits, in order to prompt discussion with healthcare workers regarding the continued necessity of catheters. Yet, in the details of the strategy, study design and implementation, it becomes clear that this project is

a multi-component intervention, with the app only being 1 part. The other components of the intervention include providing the ward feedback on the prevalence and indication of the urinary catheters, as a strategy to improve healthcare worker awareness of catheters. Additionally, the healthcare workers fill out questionnaires on the awareness of CAUTI risks on a weekly basis. Additionally, when the indication of the catheter is unclear in the records, "nurse lists", and observations, the healthcare worker is contacted to clarify the indication. These other parts of the project serve as interventions to improve healthcare worker awareness of catheter status, indications, as well as education and recurring feedback regarding urinary catheter and CAUTI risks - beyond the Catheter Check app. For these reasons, please consider a modification in the study design to allow testing of these other, more conventional interventions to reduce urinary catheter use separately - to better understand the impact of these different types of interventions. These could be tested in a step-wedge design in the programs, testing phases of A) education and feedback of urinary catheter use and indications to HCWs, B) Patient App alone, (with some programs doing phase A first, others doing phase B first), then phase C being a combination of education/ feedback to HCWs as well as app for patients. The addition of Pain Score app is an interesting strategy to motivate improved use of the app. This program is not evaluating the impact of the Pain Score check component of the application - this seems like a lost opportunity to study its impact as well as potential unintended consequences, such as higher and potentially inappropriate opioid prescribing - which some literature suggests was a consequence of mandatory "pain score" assessments in the United States for inpatients.

2.3 Project group

E	VG	G	S	U
X				

Consider the following factors:
- relevant expertise;
- familiarity with area in question;
- prior activities and products

The team appears quite appropriate with experience in Medicine as well as specialization of Infectious Disease, and expertise in App development and interventions to motivate changes in behavior. The development and prior testing of the application seems rigorous, particularly with involvement of patients and caregivers.

2.4 Feasibility

E	VG	G	S	U
X				

Consider the following factors:
- will it be possible to achieve the objective(s) using this strategy?
- availability of facilities/staff;
- realistic phasing and timetable.
- factors which may positively or negatively impact the feasibility;
- collaboration with relevant stakeholders and intermediate target groups.

The project, as described, appears quite feasible, with a realistic timetable for kick-off, implementation, and evaluation, but please consider the comments in the "Strategy" section of this evaluation to for modifications to consider to better understand the impact of several components of this intervention. Currently, the published literature on interventions to prevent CAUTI is hampered by studying primarily multi-component interventions as a bundle, with the bundles varying from project to project, and getting increasingly resource-intensive over time, with little assessment of impact of education versus intense/frequent/real-time feedback versus new technologies. Of note, I am not familiar with how comfortable patients are in general in these proposed hospitals with initiating conversations with nurses and doctors to question the necessity of the catheter - this is important to consider, as it varies by culture of the country, as well as possibly by gender, and age group. It may also be good to consider some type of measure of patient comfort level with initiating these types of conversations at baseline, as it will influence the impact of the App, particularly if in the future you are interested in implementing in different settings and countries.

2.5 Overall quality assessment

E	VG	G	S	U
	X			

This is a very interesting project, with a novel and intriguing component of educating and providing reminders to patients about the risks/benefits of indwelling urinary catheters, in order to prompt discussions with healthcare workers regarding catheter necessity. The individual components are all anticipated to have an impact on urinary catheter awareness by healthcare workers, to prompt consideration of removal - so consider revising the study design to allow an assessment of the impact of the various components (as described in Strategy feedback). The application was well written as read in English, with only a few minor sentences that were unclear in meaning. T he authors took great care in the description of the problem to be addressed, the intervention components to be studied, implementation, evaluation, informed consent, patient privacy and data protections. I look forward to hearing about this project in the future.

3. Budget

Legenda: TH (Too high), R (realistic), TL (too low)

3.1 Budget

TH	R	TL
	X	

The FTE needs described are realistic, as is the budget for the components described in materials, equipment, implementation costs.
