# Supplementary material for: A Smartphone App for Engaging Patients With Catheter-Associated Urinary Tract Infections: Protocol for an Interrupted Time-Series Analysis
Source: JMIR Res Protoc. 2021 Mar 23;10(3):e28314. doi: 10.2196/28314 (PMC8086777; doi:10.2196/28314)
Supplement: Multimedia Appendix 3 [file resprot_v10i3e28314_app3.pdf]

Subsidieprogramma / Subsidy programme	:	**Infectieziektebestrijding 2014-2017**
Dossiernummer / Dossier number	:	**50-52200-98-559**
Aanvrager / applicant	:	**Prof. dr. N.H. Chavannes MD PhD**
Projecttitel / Project title	:	**Reducing the risk of catheter-associated urinary tract infections via a smartphone application for patients – Participatient**
Beoordelingscode / Assessment code	:	**B.2017.01BD2**

1. General

Please provide considerations which contributed to your assessment of each of the assessment criteria.

Note that we will send your assessment to the applicant in anonymised form. He or she will then have an opportunity to respond. We would therefore urge you to avoid making any references to yourself in your reviewer's report.

2. Criteria

Legenda: E (Excellent,), VG (Very good), G (Good), S (Sufficient), U (Unsatisfactory)

2.1 Objective, problem definition and assignment

E	VG	G	S	U
			X	

Objective, problem definition and assignment Consider the following factors:
- how clear and specific the objective is;
- how clear and verifiable the problem definition/assignment is and whether it is consistent with the objective;
- the value added to existing knowledge or practice;
- the theoretical or empirical evidence presented in support of the problem definition/assignment.

The applicants make a reasonable case for the need to address catheter associated urinary tract infections (CAUTI) in the hospital setting, although it would have been better to provide more specific information (i.e. the proportion of UTI that are CA, rather than just 'most'). The use of a smartphone app to help empower patients sounds novel and appealing, but unfortunately the applicants do not provide sufficient evidence to support this approach. They describe this as an implementation project, which I take to be about understanding and evaluation attempts to increase uptake of an existing intervention with proven effectiveness. This does not seem to be the case with their intervention, so I believe what they are actually talking about is a study to evaluate the effectiveness of this complex intervention. However, I would have liked to see more data from their pilot work - what proportion of patients on relative wards have a smart phone, what proportion downloaded the app, what proportion used it, etc? If these data are not available then perhaps a small feasibility study is needed first. The primary aim is to reduce 'inappropriate' catheter use. Why not aim to reduce CAUTI, which is the problem that is described as needing to be addressed?

2.2 Strategy

E	VG	G	S	U
			X	

Consider the following factors:
- clarity;
- adequacy in terms of problem definition/assignment;
- adequacy of chosen methods and analyses;
- adequate random sampling and power calculation;
- the way in which the strategy reflects the factors gender, age, ethnicity and/or other characteristics relevant to the objective;
- degree of collaboration with intermediate and/or ultimate target group (the patient/client perspective).

With an implementation project:
- analysis of the context in which implementation is to take place;
- extent to which target groups are mentioned;
- a good mix of implementation activities;
- analysis of factors facilitating or hampering those activities;
- participation of stakeholders;
- prospect of structural incorporation in system;

- adequacy of process and effect evaluation design.

I am not convinced by the argument for not randomising. If this were in fact a study about implementing an intervention whose effectiveness had already previously been demonstrated in an RCT, then I think an interrupted time series (ITS) approach would be reasonable. However, this is not the case. There is no data on the effectiveness of this intervention and therefore I think the optimal design would clearly be an RCT. As this is a complex intervention, with posters and staff training as well as the app (something which does not seem to be fully recognised by the applicants), and there would be a risk of contamination, then a cluster randomised design (at ward level?) would probably be required. An ITS design is clearly inferior, especially when the primary outcome is somewhat subjective (there is a lack of detail about how appropriateness will be judged, I imagine there is at least a degree of subjectivity in the assessment). An ITS approach might be more reasonable if the primary outcome were more objective, but an RCT would always be preferable if possible, and the applicants do not make a good case for why an RCT is not possible.

2.3 Project group

E	VG	G	S	U
X				

Consider the following factors:
- relevant expertise;
- familiarity with area in question;
- prior activities and products

The project group seem to have the relevant expertise and experience

2.4 Feasibility

E	VG	G	S	U
		X		

Consider the following factors:
- will it be possible to achieve the objective(s) using this strategy?
- availability of facilities/staff;
- realistic phasing and timetable.
- factors which may positively or negatively impact the feasibility;
- collaboration with relevant stakeholders and intermediate target groups.

There does not seem like much time for setting up and training the sites or conducting the data cleaning, analysis and study write-up. I also have some concerns about whether enough research nurse time is included.

2.5 Overall quality assessment

E	VG	G	S	U
			X	

I think this is a novel and interesting idea. The problem of CAUTI in hospitals is clearly important, and the applicants make a good case for encouraging patient empowerment rather than further attempts as staff education. However, although the idea of an app as a central part of a complex intervention is appealing, I don't feel the applicants have presented enough feasibility / pilot data to make a convincing case for an effectiveness evaluation, let alone an implementation study. Furthermore, I think the team should be aiming to reduce CAUTI, rather than just 'inappropriate' catheter use, and I am concerned that the proposed ITS design will not be sufficiently robust to answer the study questions. The team appear to be very experienced.

3. Budget

Legenda: TH (Too high), R (realistic), TL (too low)

3.1 Budget

TH	R	TL
		X

This is a relatively large project, which would require considerable training and support at study sites. I am concerned that the resourcing proposed is not sufficient for this.
